# *Andes Virus* and First Case Report of Bermejo Virus Causing Fatal Pulmonary Syndrome

**DOI:** 10.3201/eid0804.010300

**Published:** 2002-04

**Authors:** Paula Padula, Marcelo González Della Valle, María Garcia Alai, Pedro Cortada, Mario Villagra, Alberto Gianella

**Affiliations:** * Instituto Nacional de Enfermedades Infecciosas, Buenos Aires, Argentina; † Hospital San Vicente de Paul Orán, Salta, Argentina; ‡ Centro Nacional de Enfermedades Tropicales, Santa Cruz, Bolivia; § Ministerio de Salud, La Paz, Bolivia.

**Keywords:** Hantavirus, HPS, Bolivia, Bermejo virus, Andes Nort virus

## Abstract

Two suspected hantavirus pulmonary syndrome (HPS) cases from Bolivia were confirmed by enzyme-linked immunosorbent assay. (ELISA)-ANDES was performed using N-Andes recombinant antigen serology in May and July 2000. Clot RNAs from the two patients were subjected to reverse transcription–polymerase chain reaction (PCR) amplification and sequencing. We describe two characterized cases of HPS. One was caused by infection with Bermejo virus and the other with Andes Nort viral lineage, both previously obtained from *Oligoryzomys* species. This is the first report of molecular identification of a human hantavirus associated with Bermejo virus.

Hantaviruses cause hemorragic fever with renal syndrome (HFRS) in Eurasia and hantavirus pulmonary syndrome (HPS) in the Americas. Infection occurs primarily by the respiratory route via aerosolized virus in rodent excreta. Field investigations have identified sigmodontine rodents as the host of the respiratory hantaviruses. HPS cases have been reported in several American countries during the last 8 years including Argentina, Bolivia, Brazil, Canada, Chile, Panama, Paraguay, United States, and Uruguay (*1*–*5*). *Andes virus* (ANDV) has been responsible for most of the >400 cases recorded in Argentina, Chile, and Uruguay; *Oligoryzomys* is the predominant rodent carrier. A different genus, *Calomys laucha,* was responsible for most cases in Paraguay associated with *Laguna Negra virus* (LNV) (*6*). Several additional hantaviruses have been detected in rodents in the Americas but have yet to be associated with human disease. Cases of HPS shown to be caused by ANDV (Andes Nort lineage) have been reported in northern Argentina in the Salta and Jujuy Provinces. No cases have been reported previously in Bolivia except in a Chilean HPS patient who was suspected of having acquired the infection during extensive travel in Bolivia prior to onset of illness in 1997. The viral characterization revealed an LNV variant that was 15% at the nucleotide level and identical at the deduced amino acid level relative to LNV (*6*). Molecular techniques have aided in the testing of Bolivian specimens from a museum collection and allowed the identification of a hantavirus named *Rio Mamore virus* (RIOM) from *Oligoryzomys microtis*. RIOMV has not been associated with human cases yet (*7*).

In 2000, a total of six male patients in Bolivia (15 to 49 years) were serologically confirmed to have HPS; five died. Five out of the six cases occurred between April and July; the sixth case occurred in November. All the patients lived and worked in rural areas in a 70-km ratio around Bermejo, although it was not clear whether one of them (Patient 2) was infected in Argentina or in Bolivia. In addition, two HPS cases were previously reported in Bolivia, one in June 1998 and the other in October 1999.

## Case Reports

### Patient 1

On May 18, 2000, a 49-year-old man from Bermejo, Bolivia, who worked as a woodcutter in San Telmo, had a onset of HPS symptoms. Seven days later he was hospitalized at the San Vicente de Paul Hospital and his case was confirmed by enzyme-linked immunosorbent assay (ELISA)-ANDES specific immunoglobulin(Ig) M and immunoglobulin(Ig) G test (*8*). The patient survived. He had respiratory distress with bilateral interstitial infiltrates. Initial laboratory results were as follows: serum creatinine 1.4 mg/dL, platelets 50,000/μL, hematocrit 46%, arterial O_2_ pressure (PaO_2_) 50mm Hg, arterial CO_2_ pressure (PaCO_2_) 32mm Hg, leukocytes 6,700 /mm^3^, urea 0.36 g/L, bilirubin 0.52. mg/100 mL. Laboratory results 4 days after he was hospitalized were as follows: hematocrit 46%, 46.8%, 48%, 43%; platelets/mm^3^ 50,000, 32,000, 30,000, and 128,000; and white blood cells/mm^3^ 6,700, 15,400, 20,700, and 18,300.

### Patient 2

In July 2000, a 20-year-old man from Bermejo was admitted to the San Vicente de Paul Hospital 4 days after symptoms began; a preliminary diagnosis of HPS was made. The patient’s condition deteriorated rapidly, showing respiratory compromise with bilateral interstitial infiltrates and renal compromise with oliguria. Laboratory results were as follows: serum creatinine 2.3 mg/dL, lactic dehydrogenase (LDH) 572 IU/L, platelets 26,000 /μL, hematocrit 71%, PaO_2_ 73, PaCO_2_ 35, and leukocytes 64,300 /μL. The patient died the same day he was hospitalized. HPS was confirmed by ELISA-ANDES-specific IgM and IgG test. Although the patient lived in Bolivia, he worked as a muleteer in Rio Blanco River in Salta Province, Argentina for 2 months and came back to Bolivia 3 weeks before his illness.

## The Study

Clot samples from the two patients were used to prepare RNA with an RNA matrix (RNAid kit, BIO101, La Jolla, CA). The RNA was subjected to reverse transcription-polymerase chain reaction (PCR) amplification by nested or heminested reactions. Synthesized DNA products were separated on agarose gels, gel-purified, and directly sequenced with an ABI 377 sequencer. Three cDNA products were produced, allowing generation of nucleotide (nt) sequences of 289 nt in length for an S segment region encoding the most conserved region of the nucleoprotein (N), and 243 nt and 226 nt in length for two M segment regions of G1 and G2, respectively. The nucleotide sequences were determined and compared with known hantaviruses (Table). Sequence identity differences of 3.3% and 1.2% were seen at the nucleotide and amino acid levels respectively, when the G1 fragment sequence of Patient 1 was compared with those of other viral sequences associated with cases in North Argentina. Genetic analysis showed that Patient 1’s viral sequence belonged to the Andes Nort lineage characterized previously (*2*,*4*). The hantavirus in Patient 2 was clearly identified as Bermejo virus, previously detected in one *O. chacoensis* captured in Orán City, Salta (*2*). We ruled out laboratory DNA contamination since this was the first available sample of RNA virus in our hands. Viral G1 fragment sequence from this patient showed 100% nucleotide identity compared with the Bermejo virus. Nucleotide and amino acid differences of 13.2% to 24.3% and 3.7% to 8.6%, respectively, were seen when the G1 viral fragment from Patient 2 was related with the more distantly ANDV and closely related lineages including Andes Nort, Andes Sout, and the three different Andes Cent lineages (*4*). To provide more information on the genetics of Bermejo virus, a highly variable fragment of the M segment (nt 88 to 442) was amplified and sequenced (GenBank accession number AF442564). Nucleotide and amino acid comparison of this fragment between Patient 2 and Andes Nort lineage showed a divergence of 17.2% and 9.3%, respectively. Additionally, a divergence of 6.2% and 0% in the S conserved fragment of Patient 2 and Andes Nort lineage at the nucleotide and amino acid level, respectively, was observed. Unfortunately, homologous fragment sequences from Bermejo virus were not available. Maximum parsimony phylogenetic analysis of the G1 fragment sequences of the two patients and other published hantavirus sequences showed the expected clusters between the viral sequence detected in Patient 1 and Andes Nort lineage virus and the viral sequence in Patient 2 and Bermejo virus (data not shown).

**Table:** Nucleotide and amino acid sequence^a^ divergence comparisons between the 2 Bolivian patients and other hantavirus pulmonary syndrome (HPS)-related viruses of 2 fragments of the M segment^b^, and 1 fragment of the S segment^c^

	Patient 1			Patient 2			ANDV Nort (Argentina)			Bermejo virus (Argentina)			RIOM (Bolivia)			HTN007 (Peru)			LN (Paraguay)		
	N	G1	G2	N	G1	G2	N	G1	G2	N	G1	G2	N	G1	G2	N	G1	G2	N	G1	G2
Patient 1	—	—	—	5,9	19,8	NA^d^	1,4	3,3	3,1	NA	19,8	15,5	12,8	NA	NA	14,2	NA	NA	15,6	24,3	17,7
Patient 2	0	8,6	NA	—	—	—	6,2	21	NA	NA	0	NA	14,2	NA	NA	14,9	NA	NA	17,3	29,2	NA
ANDV Nort (Argentina)	0	1,2	1,3	0	7,4	NA	—	—	—	NA	21	14,2	13,2	NA	NA	14,2	NA	NA	16,6	23,1	17,7
Bermejo virus (Argentina)	NA	8,6	2,7	NA	0	NA	NA	7,4	1,3	—	—		NA	NA	NA	NA	NA	NA	NA	29,2	19
RIOM (Bolivia)	11,5	NA	NA	11,5	NA	NA	11,5	NA	NA	NA	NA	NA	—	—	—	5,5	NA	NA	9	NA	NA
HTN007 (Peru)	11,5	NA	NA	11,5	NA	NA	11,5	NA	NA	NA	NA	NA	2,1	NA	NA	—	—	—	11,1	NA	NA
LN (Paraguay)	12,5	14,8	8	12,5	17,3	NA	12,5	13,6	6,7	NA	17,3	5,3	2,1	NA	NA	4,2	NA	NA	—	—	—

^a^GenBank accesion numbers for the nucleotide sequences are: Patient 1 N:AF442561 G1:AF442559 G2:AF442560; Patient 2 N:AF442563 G1:AF442562; values above dashes are nucleotide sequence divergence and those below dashes are amino acid sequence divergence. The comparison analysis was performed using NALIGN and PALIGN programs from PCGENE 6.8 software from Intelligenetics Inc. (Mountain View CA). ^b^M segment G1 (nt 1735 to 1977); M segment G2 (nt 2718 to 2943). ^c^S segment N (nt 48 to 336) ^d^NA= not available

In previous studies, *O. chacoensis* was tentatively implicated as the predominant rodent reservoir for Bermejo virus; however, the sample size was too small since Bermejo virus was identified in only 1 rodent (*2*). Specific cases of HPS were not linked to the occurrence of the Bermejo virus-infected rodent at the presumed site of infection. Moreover, Andes Nort lineage was previously characterized in one rodent (*O. flavescens)*, two rodents (*O. chacoensis)* (*9*), and two rodents (*O. longicaudatus)* (*2*). Whether the viruses responsible for the infection in our report were harbored by any particular *Oligoryzomys* species is now being investigated.

Molecular and epidemiologic data showed the presence of Andes Nort lineage circulating in Bolivia. Considering that the incubation period was estimated to be 19.5 days (*4*) and since Patient 2 returned from Argentina 21 days before the onset of disease, in which country the infection occurred was unclear. This is the first report of molecular identification of a human hantavirus associated with Bermejo virus.
